# Supplementary material for: DMRforPairs: identifying Differentially Methylated Regions between unique samples using array based methylation profiles
Source: BMC Bioinformatics. 2014 May 15;15:141. doi: 10.1186/1471-2105-15-141 (PMC4046028; doi:10.1186/1471-2105-15-141)
Supplement: Additional file 2 — DMRforPairs output for the comparison of A431-MCF7 and NA17018-NA17105. Please start from the HTML files in each folder. Available via the BMC Bioinformatics website. [file 1471-2105-15-141-S2.zip › 1394847754114233_MOESM2_ESM/A431_MCF7/figures/33929.pdf]

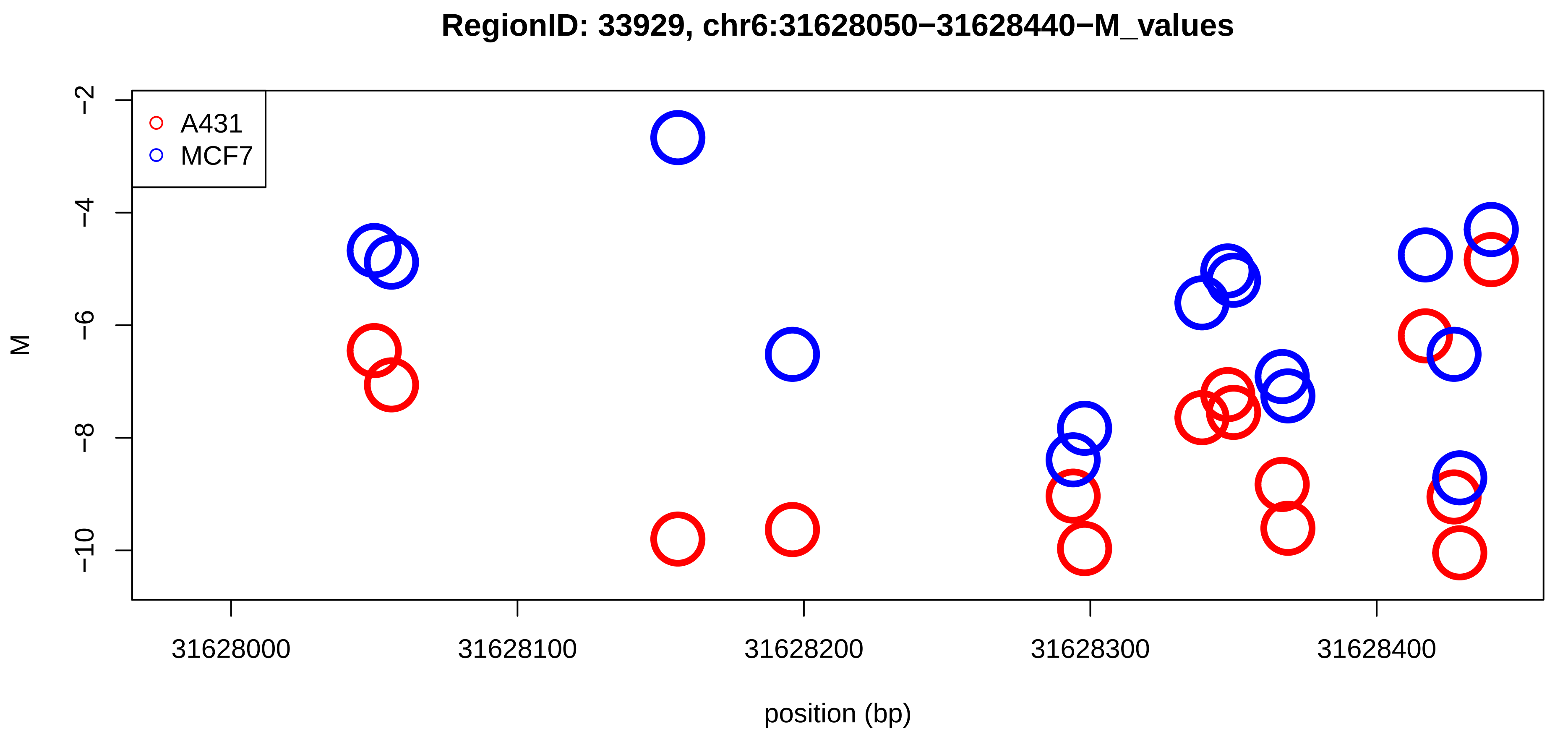

RegionID: 33929, chr6:31628050−31628440−M_values
A431
MCF7
M
−2
−4
−6
−8
−10
31628000
31628100
31628200
31628300
31628400
position (bp)

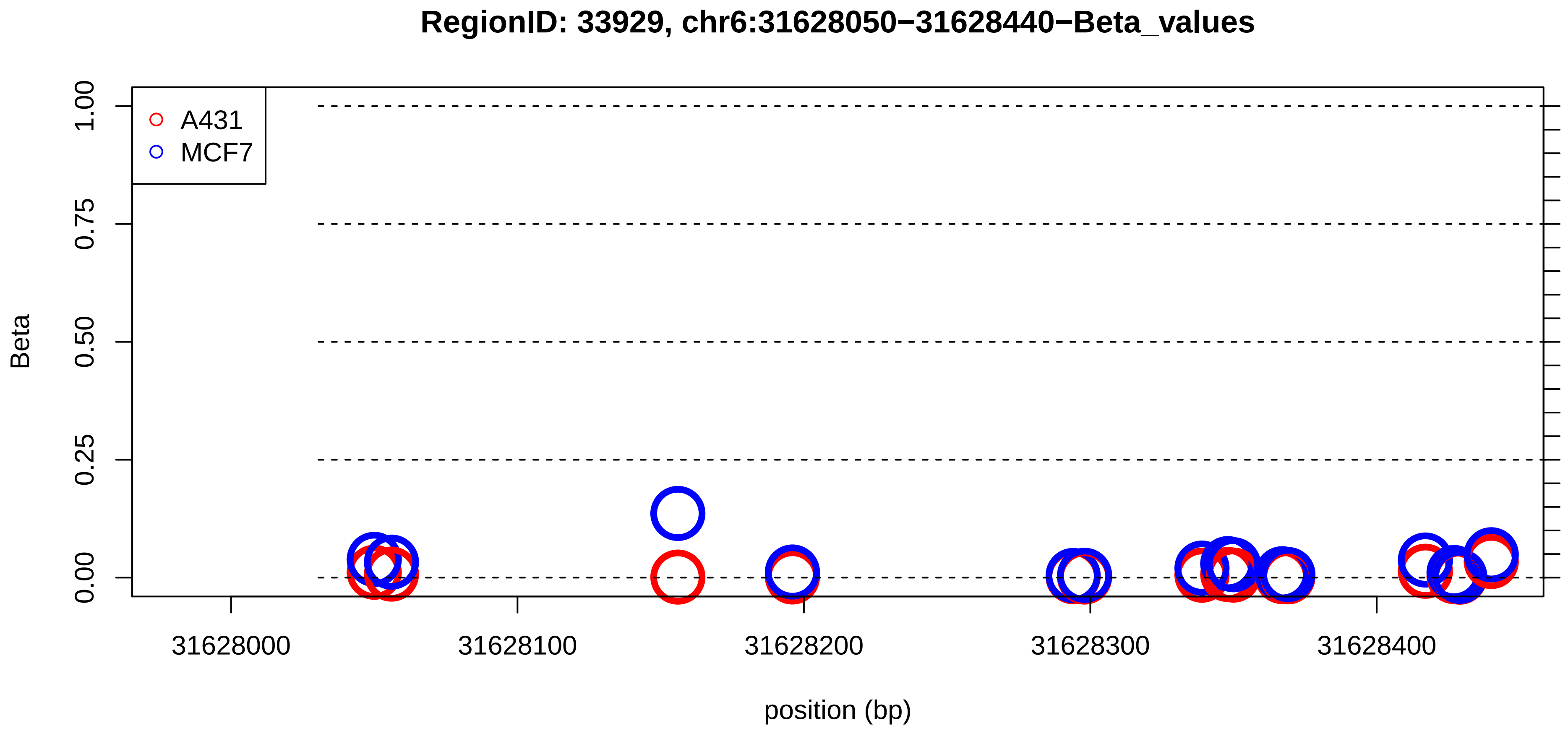

RegionID: 33929, chr6:31628050−31628440−Beta_values
A431
MCF7
Beta
0.00
0.25
0.50
0.75
1.00
31628000
31628100
31628200
31628300
31628400
position (bp)

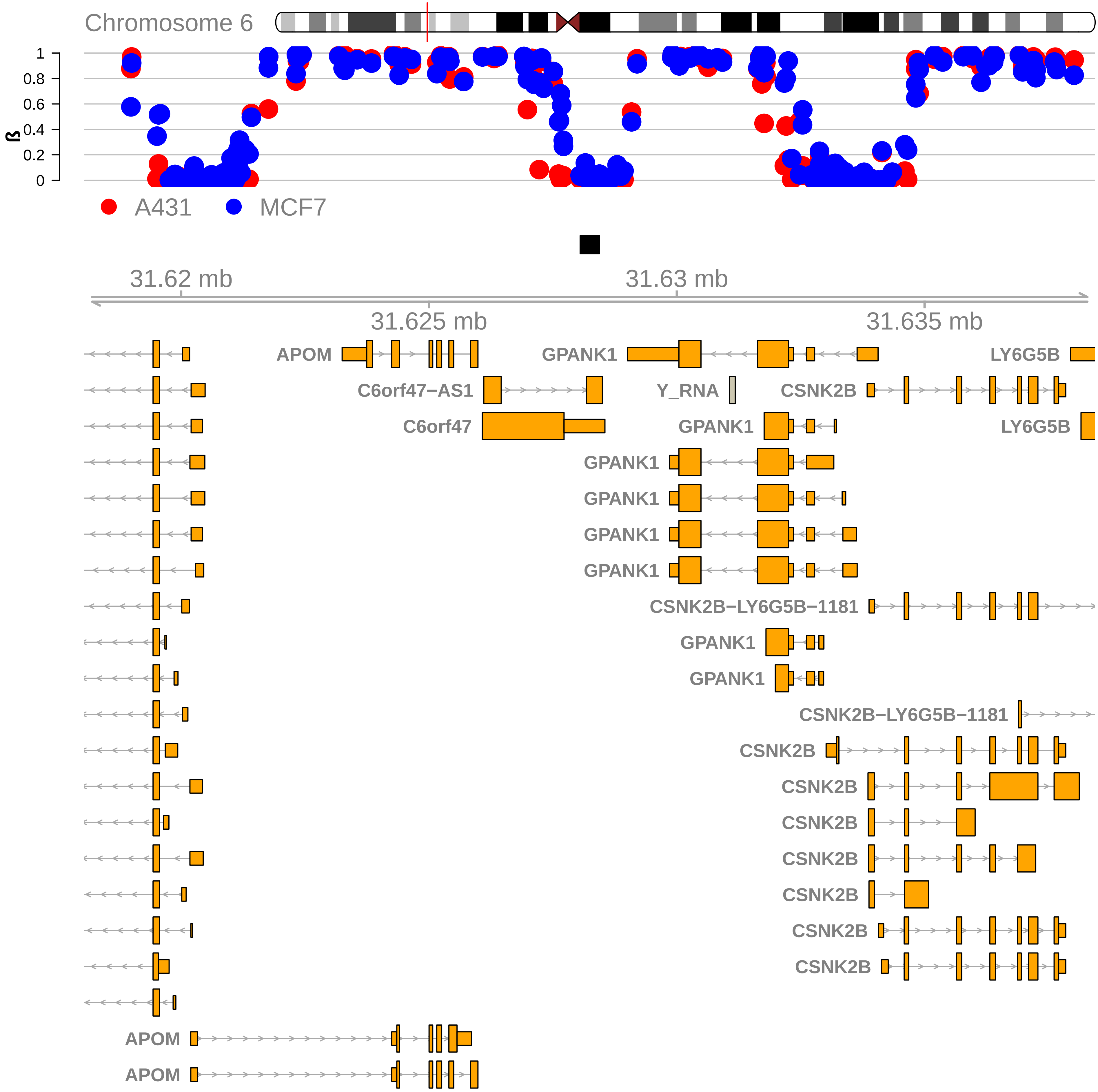

Chromosome 6
ß
1
0.8
0.6
0.4
0.2
0
A431
MCF7
31.62 mb
31.625 mb
31.63 mb
31.635 mb
APOM
GPANK1
LY6G5B
C6orf47−AS1
Y_RNA
CSNK2B
C6orf47
GPANK1
LY6G5B
GPANK1
GPANK1
GPANK1
GPANK1
CSNK2B−LY6G5B−1181
GPANK1
GPANK1
CSNK2B−LY6G5B−1181
CSNK2B
CSNK2B
CSNK2B
CSNK2B
CSNK2B
CSNK2B
CSNK2B
APOM
APOM
